# Uncovering LiH Triggered Thermal Runaway Mechanism of a High‐Energy LiNi_0.5_Co_0.2_Mn_0.3_O_2_/Graphite Pouch Cell

**DOI:** 10.1002/advs.202100676

**Published:** 2021-05-24

**Authors:** Lang Huang, Gaojie Xu, Xiaofan Du, Jiedong Li, Bin Xie, Haisheng Liu, Pengxian Han, Shanmu Dong, Guanglei Cui, Liquan Chen

**Affiliations:** ^1^ Qingdao Industrial Energy Storage Research Institute Qingdao Institute of Bioenergy and Bioprocess Technology Chinese Academy of Sciences No. 189 Songling Road Qingdao 266101 China; ^2^ Center of Materials Science and Optoelectronics Engineering University of Chinese Academy of Sciences No. 19A Yuquan Road Beijing 100049 China; ^3^ Key Laboratory for Renewable Energy Beijing Key Laboratory for New Energy Materials and Devices Beijing National Laboratory for Condensed Matter Physics Institute of Physics Chinese Academy of Sciences Beijing 100190 China

**Keywords:** batteries, heat generation, thermal safety

## Abstract

The continuous energy density increase of lithium ion batteries (LIBs) inevitably accompanies with the rising of safety concerns. Here, the thermal runaway characteristics of a high‐energy 5 Ah LiNi_0.5_Co_0.2_Mn_0.3_O_2_/graphite pouch cell using a thermally stable dual‐salt electrolyte are analyzed. The existence of LiH in the graphite anode side is innovatively identified in this study, and the LiH/electrolyte exothermic reactions and H_2_ migration from anode to cathode side are proved to contribute on triggering the thermal runaway of the pouch cell, while the phase transformation of lithiated graphite anode and the O_2_‐releasing from cathode are just accelerating factors for thermal runaway. In addition, heat determination during cycling at two boundary scenarios of adiabatic and isothermal environment clearly states the necessity of designing an efficient and smart battery thermal management system for avoiding heat accumulation. These findings will shed promising lights on thermal runaway route map depiction and thermal runaway prevention, as well as formulation of electrolyte for high energy safer LIBs.

## Graphical abstract

During elevated temperatures, pouch cell starts the heat accumulation process from the anode/electrolyte interfacial reactions, accompanied with a large amount of H_2_ producing. The anode‐liberated H_2_ then migrates to cathode side, cross‐interacting with the delithiated cathode to induce O_2_ releasing and tremendous heat generation, which further accelerates the exothermic chain reactions and leads to severe thermal runaway.

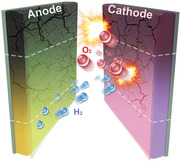

## 1 Introduction

This decade has witnessed the increasing challenges coming from petrochemical energy crisis and environmental pollution. Hence, the demand for developing advanced and renewable energy technology with clean processes and high efficiency is becoming more and more necessary. Great efforts toward promoting alternative renewable energy resources (such as solar, wind, photovoltaic, and tidal energy, etc.) have been devoted.^[^ ^1^ ^]^ The intermittent nature of these renewable energy resources facilitates the booming of varied energy storage systems. Due to their advantages like high energy density, low maintenance, and longevity, the promising energy storage system of Li‐ion batteries (LIBs) have been widely used in varied fields ranging from small portable electronics to large‐scale electric vehicles.^[^ ^2^ ^]^

Recently, the on‐going “endurance mileage” anxiety has stimulated the energy density increase of LIBs, and great efforts has been made on understanding the inherent electrochemistry as well as developing advanced material systems. However, the energy density increase of LIBs inevitably accompanies the rising of safety concerns.^[^ ^3^, ^4^, ^5^ ^]^ Compulsory and strict testing standards are applied for LIBs before their entrance into market, and the thermal characteristics research of LIBs is kept attracting widely attentions. At present, varied testing approaches, such as accelerating rate calorimetry (ARC),^[^ ^6^, ^7^ ^]^ differential scanning calorimetry (DSC),^[^ ^8^ ^]^ C80 microcalorimeter,^[^ ^9^ ^]^ and isothermal microcalorimetry (IMC),^[^ ^10^ ^]^ etc., have been employed to investigate thermal characteristics and thermal runaway mechanism of LIBs. Conventionally, the thermal runaway mechanism of LIBs is demonstrated to be associated with a series of exothermic chain reactions, including decomposition of solid electrolyte interface (SEI) layer, anode/electrolyte reactions, self‐decomposition of electrolyte, and cathode/electrolyte reactions, etc.^[^ ^11^, ^12^ ^]^ However, Prof. M. Ouyang et al. revealed that the oxygen released from nickel‐manganese‐cobalt cathode will be consumed by the lithiated anode with great heat generation, triggering the thermal runaway of LIBs.^[^ ^7^ ^]^ Differently, by analyzing the released gas, Galushkin et al. propose that the powerful exothermic reaction from recombination of atomic hydrogen accumulated at anode graphite will contribute to the initiation of thermal runaway of LIBs.^[^ ^13^ ^]^ It is noted here that, battery thermal runaway will occur at any state of charge (SOC) in practical cases. But in most of previous reports, the thermal runaway mechanisms are always deciphered at high SOC. Until now, due to the complexity of exothermic chain reactions inside LIBs and the limitation of the existing testing approaches for thermal safety study, it is still difficult to obtain a clear and accurate thermal runaway route map depicting the rooted interactions among cathode, anode, electrolyte, and separator.

Obviously, during the hazardous thermal runaway (smoke, fire, and even explosion) of LIBs, electrolyte is almost involved in every exothermic chain reaction.^[^ ^3^, ^14^ ^]^ As the “blood” of the LIBs, the organic electrolyte has a crucial influence on the electrochemical and inherent thermal safety of LIBs. Lithium hexafluorophosphate (LiPF_6_) with well‐balanced properties is always adopted as the main conducting lithium salt for the widely commercialized carbonate‐based electrolytes. However, the thermally unstable and moisture sensitive LiPF_6_ is susceptible to generate undesired reactive species, such as HF and POF_3_, inducing the destruction of electrode/electrolyte interface layers and the transition metals dissolution from cathode.^[^ ^15^, ^16^ ^]^ Hence, tremendous efforts have been devoted to design and synthesize alternative lithium salts.^[^ ^15^, ^17^ ^]^ Wherein, thermally stable and highly conductive lithium imides of lithium bis(trifluoromethanesulfonyl) imide (LiTFSI) and lithium bis(fluorosulfonyl) imide (LiFSI) have aroused great interests. But, both LiTFSI and LiFSI easily cause corrosion of Al current collector at high voltages exceeding 4 V.^[^ ^18^ ^]^ Formulating high cost concentrated electrolyte will effectively prohibit LiFSI‐induced Al corrosion, but, the LIB still undergoes thermal runaway due to the strong heat‐releasing reaction between LiFSI salt and lithiated graphite.^[^ ^11^ ^]^ Another economic way to suppress Al corrosion is to formulate blended‐salt electrolytes by mixing LiTFSI or LiFSI with lithium difluoro(oxalate) borate (LiDFOB) or lithium bis(oxalato)borate (LiBOB). Recently, blended‐salt electrolytes showing synergistic effects have achieved great progress in the burgeoning field of next‐generation lithium batteries. Nevertheless, to the best of our knowledge, the electrochemical and thermal safety evaluation of blended‐salt electrolytes in large format and large capacity LIBs are seldom reported.

Here, we conduct electrochemical and thermal safety study of a pouch‐type 5 Ah LiNi_0.5_Co_0.2_Mn_0.3_O_2_/graphite (NCM523/G) battery with a dual‐salt electrolyte, which is formulated by dissolving LiTFSI and LiDFOB lithium salts in carbonate solvents of ethylene carbonate (EC), propylene carbonate (PC), and ethyl methyl carbonate (EMC). In specific, the electrochemical performances of 5 Ah NCM523/G pouch cell are investigated over a wide temperature range (−40–60 °C). More importantly, varied advanced characterization techniques (such as temperature‐resolved X‐ray diffraction (XRD), ARC, on‐line titration gas analysis system) are used to elucidate the thermal compatibility of battery materials disassembled from pouch cell with different SOC (100% and 0%). We innovatively propose that the LiH induced heat generation and the H_2_ release at anode side migrating to cathode side is the rooted thermal runaway trigger of this NCM523/G pouch cell, while the phase transformation of lithiated graphite anode and the O_2_‐releasing by delithiated NCM523 cathode are just accelerating factors for thermal runaway. In addition, to state the importance of designing an efficient and smart battery thermal management system, heat generation of 5 Ah NCM523/G pouch cell during charge–discharge processes is determined at both adiabatic (ARC) and isothermal (IMC) conditions. This study provides a deeper insight for understanding of the inherent mechanism of thermal runaway of LIBs, and lights the way to advanced design philosophy of next generation safer LIBs.

## 2 Results and Discussion

### 2.1 Electrochemical Performances over a Wide Temperature Range

**Figure 1:**
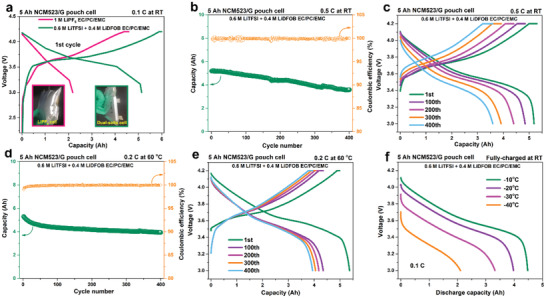
(a) The 1^st^ formation cycle of 5 Ah NCM523/G pouch cell using 1 M LiPF_6_ EC/PC/EMC and a dual‐salt electrolyte of 0.6 M LiTFSI + 0.4 M LiDFOB EC/PC/EMC. The electrochemical cycling performances of 5 Ah NCM523/G pouch cell using the dual‐salt electrolyte, (b,c) at RT and (d,e) at 60°C, respectively. (f) The low temperature discharge curves of 5 Ah NCM523/G pouch cell using the dual‐salt electrolyte. Insets in (a) are the photographs of pouch cells after the formation process.

Undoubtedly, LIBs operating over a wide temperature range presents great importance in practical applications, such as those in electric vehicles, space, and military missions. For wide temperature LIBs, the most challenging work is to get a compromise between subzero temperature performance and high temperature performance. The most direct and efficient strategy is formulating wide temperature electrolyte by dissolving thermally stable lithium salts in low melting point and high boiling point solvents with low viscosity.^[^ ^5^, ^19^ ^]^ Herein, a dual‐salt electrolyte of 0.6 M LiTFSI + 0.4 M LiDFOB EC/PC/EMC (1:1:3, by volume) is developed for 5 Ah NCM523/G pouch cell. Wherein, carbonate solvents possessing low melting point (PC, *T*_m_ = −48.8 °C; EMC, *T*_m_ = −53 °C) and high boiling point (EC, *T*_b_ = 243 °C; PC, *T*_b_ = 242 °C) are selected for formulating the wide temperature range electrolyte. In the first formation cycle at room temperature (RT), 5 Ah NCM523/G pouch cell with the dual‐salt electrolyte demonstrated high initial Coulombic efficiency of 85.8% (5.14/5.99 Ah) than that (47.6%, 2.18/4.58 Ah) of the pouch cell with 1 m LiPF_6_ EC/PC/EMC (1:1:3, by volume) (**Figure** **1a**). Moreover, severe gas swelling of pouch cell with 1 m LiPF_6_ EC/PC/EMC is observed (Left inset in Figure 1a), due to the PC‐induced formation of unstable SEI layer on graphite anode.^[^ ^20^ ^]^ As a sharp contrast, pouch cell with the dual‐salt electrolyte shows no obvious swelling (Right inset in Figure 1a), benefiting from the formation of stable SEI layer by LiDFOB salt.^[^ ^21^ ^]^ Therefore, 5 Ah NCM523/G pouch cell with the dual‐salt electrolyte is employed for the following electrochemical and thermal safety study.

After the vacuum degassing procedure of formation process, the determined gravimetric energy density of 5 Ah NCM523/G pouch cell with dual‐salt electrolyte is highly up to 208.8 Wh kg^−1^. At RT at 0.5 C rate for 400 cycles, the pouch cell presents a high average Coulombic efficiency of 99.88% and delivers a discharge capacity retention of 92.72%, 84.10%, 74.90%, and 68.77%, at the 100th, 200th, 300th, and 400th cycle, respectively (Figure 1b,c). When the temperature is increased up to 60 °C, the discharge capacity retention of the pouch cell at 0.2 C rate is 82.23%, 79.02%, 76.56%, and 74.48% at the 100th, 200th, 300^th^, and 400th cycle, respectively, with an average Coulombic efficiency reaching to 99.92% (Figure 1d,e). In subsequent, subzero temperature performance is revealed by discharging the room‐temperature fully charged 5 Ah NCM523/G pouch cell, at varied subzero temperatures of −10, −20, −30, and −40 °C, respectively (Figure 1f). The corresponding discharge capacity retention is 85.79%, 75.76%, 64.46%, and 40.81%. In general, the high‐energy 5 Ah NCM523/G pouch cell using the formulated dual‐salt electrolyte is very competent for wide temperature range applications. Moreover, it is noted that, to the best of our knowledge,^[^ ^15^ ^]^ this is the first case to evaluate the application of dual‐salt electrolytes in LIBs over a wide temperature range, which is significant for the commercialization process of dual‐salt electrolytes.

### 2.2 Thermal Runaway Feature and Mechanism

**Figure 2:**
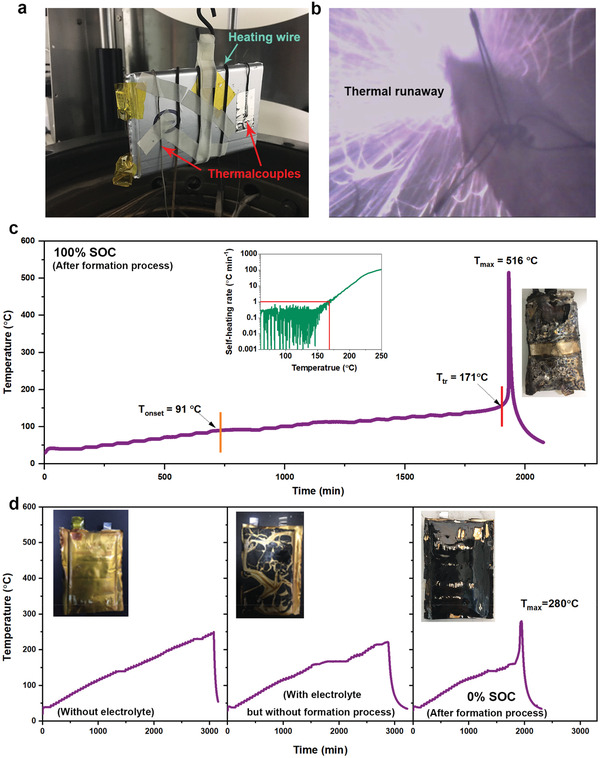
(a) The photograph of 5 Ah NCM523/G pouch cell in ARC (BTC500, HEL) for thermal runaway study. (b) The captured photograph when 5 Ah NCM523/G pouch cell gets thermal runaway in ARC. (c) Temperature profile when 5 Ah NCM523/G pouch cell (100% SOC, after formation process) is tested by the heat‐wait‐search (HWS) mode of ARC. Insets of (c) are the corresponding self‐heating rate curve and the wreck of tested pouch cell. (d) Temperature profiles when varied types of 5 Ah NCM523/G pouch cell is tested by the heat‐wait‐search (HWS) mode of ARC. Insets of (d) are the corresponding wrecks of tested pouch cells.

As a high‐energy storage reservoir, LIBs easily get thermal runaway when operated under mechanical, electrical, and thermal abuse conditions. To develop an efficient battery safety risk controlling strategy, it is necessary to obtain some critical parameters, such as onset temperature for self‐heating (*T*_onset_), self‐heating rate (SHR), thermal runaway temperature (*T*_tr_), and maximum temperature (*T*_max_), etc. Here, 5 Ah NCM523/G pouch cell using dual‐salt electrolyte is placed in the cavity of ARC (BTC500, HEL, **Figure** **2a**; and Figure S1, Supporting Information), and the typical heat‐wait‐search (HWS) mode is used to study thermal runaway features of the pouch cell. Under the HWS mode of ARC, the built‐in camera in cavity captures that smoke and flame is rapidly spurted out of the pouch cell (100% SOC, after the formation process) (Figure 2b). Obviously, the *T*_onset_, *T*_tr_ (SHR over 1 °C min^−1^ as the criteria) and *T*_max_ is determined to be 91, 171, and 516 °C, respectively (Figure 2c). After the thermal runaway process, the aluminum plastic film is severely damaged (inset in Figure 2c). In addition, the LiPF_6_ based pouch cell shows low *T*_onset_ and *T*_tr_ compared with the dual‐salt one due to the dual‐salts electrolyte presents higher thermal stability (Figure S2, Supporting Information). It is noted here, that most previous battery thermal runway investigations focus on the fully charged cell (100% SOC) because of its violent thermal runway hazardous, while the fact that fully discharged cell (0% SOC) also undergoes thermal runway is neglected. Here, to explore the rooted mechanism for the triggering of thermal runway behavior, 5 Ah NCM523/G pouch cell without dual‐salt electrolyte and 5 Ah NCM523/G pouch cell with dual‐salt electrolyte but without formation process are fabricated and tested by ARC under same conditions, and interestingly, both pouch cells do not present severe exothermic reactions related to the thermal runway of pouch cell below 250 °C (Figure 2d). However, when the 5 Ah NCM523/G pouch cell with dual‐salt electrolyte is cycled for one formation cycle (0% SOC), the thermal runaway occurs under the same testing condition in ARC (*T*_onset_ = 141 °C, *T*_tr_ = 199 °C, *T*_max_ = 280 °C). These results clearly tell us that the formed interfacial layer components between the electrolyte and electrodes play a crucial role in triggering the thermal runaway process of pouch cell. Therefore, we confirm that pouch cell after formation process will go thermal runaway anyway regardless of the SOC, and pouch cell with higher SOC demonstrates faster and more severe thermal runaway process (Figure S3, Supporting Information).

**Figure 3:**
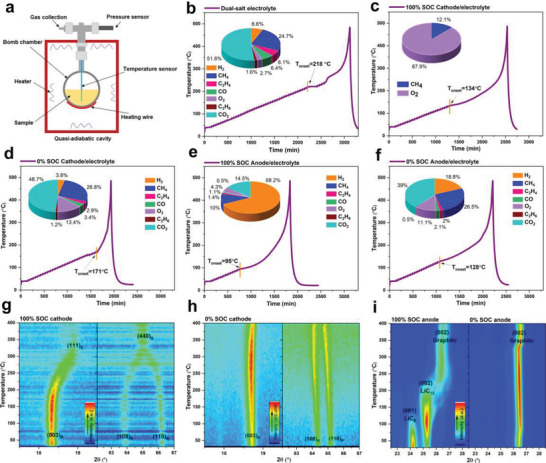
(a) Schematic illustration of the working principle of ARC (BTC130, HEL) with a small bomb chamber. Temperature profiles when (b) dual‐salt electrolyte, (c) 100% SOC cathode/electrolyte, (d) 0% SOC cathode/electrolyte, (g) 100% SOC anode/electrolyte, and (h) 0% SOC anode/electrolyte, are tested by the heat‐wait‐search (HWS) mode of ARC. The insets in (b,c,d,g,h) are the determined components percentage in collected gas after ARC testing. XRD patterns of (e), (f) cathode and (i) anode by increasing the temperature from 30 to 400°C. The anode and cathode are carefully and separately dissembled from fully charged (100% SOC) and fully discharged (0% SOC) 5 Ah NCM523/G pouch cells.

**Figure 4:**
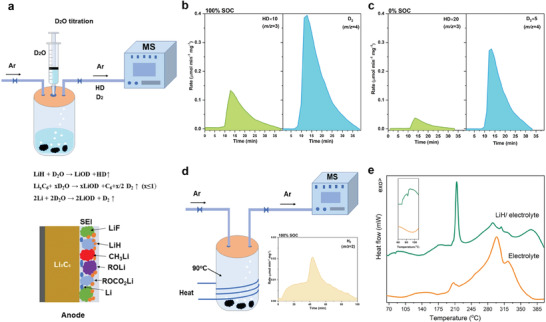
(a) Schematic illustration of delicately‐designed *on‐line* D_2_O titration gas analysis MS system. The HD and D_2_ evolution rate curve after D_2_O titration on samples of fully‐lithiated graphite (100% SOC) (b) and fully‐delithiated graphite (0% SOC) (c). (mg^−1^, divided by the sample mass for titration). The graphite anodes are carefully and separately dissembled from fully charged (100% SOC) and fully discharged (0% SOC) 5 Ah NCM523/G pouch cells. (d) Schematic illustration of *on‐line* gas analysis MS system for heating fully‐lithiated graphite (100% SOC) and the H_2_ evolution rate curve after heating (d, inset). (mg^−1^, divided by the sample mass for titration). (e) DSC curves of dual electrolyte and LiH/dual electrolyte under N_2_ atmosphere.

To decipher the rooted mechanism for triggering the thermal runaway of NCM523/G pouch cell, ARC (BTC130, HEL) equipped with a small bomb chamber is used to study the thermal compatibility of battery components (**Figure** **3a**; and Figure S4, Supporting Information). In the Ar‐filled glove box, the anode and cathode are carefully and separately dissembled from fully charged (100% SOC) and fully discharged (0% SOC) 5 Ah NCM523/G pouch cell. The as‐formulated fresh dual‐salt electrolyte presents a high *T*_onset_ of 218 °C, suggesting its high thermal stability (Figure 3b). The fully delithiated cathode/electrolyte (100% SOC) and lithiated cathode/electrolyte (0% SOC) demonstrate *T*_onset_ of 134 °C (Figure 3c) and 171 °C (Figure 3d), respectively. And the fully lithiated anode/electrolyte (100% SOC) and delithiated anode/electrolyte (0% SOC) exhibit the *T*_onset_ of 95 °C (Figure 3e) and 128 °C (Figure 3f), respectively. These results reveal that, at different SOCs, cathode/electrolyte and anode/electrolyte will inevitably go thermal runaway and anode/electrolyte is easier to get thermal runaway than cathode/electrolyte. In addition, higher thermal reactivity is presented at higher SOCs. Therefore, it is concluded here that high SOC is just the accelerating factor of battery thermal runaway, but not the root cause for triggering it. After thermal runaway of the dual‐salt electrolyte, anode/electrolyte, and cathode/electrolyte, the remained incondensable gas species in the sealed bomb chamber is collected and analyzed by the mass spectrometer (MS), respectively (insets in Figure 3b–e). Wherein, the remained gas species for thermal runaway of sole as‐formulated dual‐salt electrolyte is mainly consisted of CO_2_ (51.8%) and CH_4_ (24.7%) (insets in Figure 3b). For fully delithiated NCM523 cathode/electrolyte (100% SOC), O_2_ (87.9%) dominates the collected gas species after thermal runaway (insets in Figure 3c). Revealed by temperature‐resolved XRD equipped with an in situ heating module, when the temperature exceeds 200 °C, the fully delithiated NCM523 cathode will release O_2_ by phase transformation of layered structure ((003)_R_) to spinel structure ((111)_S_) and disordered spinel structure ((108)_R_ and (110)_R_) to the NiO‐like rock‐salt structure ((440)_S_) (Figure 3g). As an interest comparison, lithiated NCM523 cathode (0% SOC) reacting with electrolyte produces main gas species of CO_2_ (48.7%), CH_4_ (26.8%), and O_2_ (13.4%). The dramatic decrease of O_2_ percentage is possibly attributed to the well‐preserved crystal structure of lithiated NCM523 cathode (0% SOC) (Figure 3h). To further verify this, XRD patterns of the powders collected after thermal runaway of 0% and 100% SOC 5 Ah NCM523/G pouch cell are tested. It is presented that the fully delithiated NCM523 cathode (100% SOC) undergoes phase transformation and crystal structure collapse, while the main layered structure of lithiated NCM523 cathode (0% SOC) is preserved (Figure S5, Supporting Information). These results indicate that O_2_ releasing accompanying with phase transformation of delithiated NCM523 cathode at high temperature around 200 °C may aggravate the burning or explosion, but is not the rooted mechanism for triggering thermal runaway. Obviously, the rooted factor for triggering the thermal runaway still depends on anode side. After thermal runaway of fully lithiated anode/electrolyte (100% SOC), the dominated gas species is H_2_ (68.2%), while for thermal runaway of unlithiated anode/electrolyte (0% SOC), CO_2_ (39%), CH_4_ (26.5%), and H_2_ (18.8%) are the main gases determined. Except the evolution of CO_2_ and CH_4_ from thermal decomposition of electrolyte, H_2_ gas constitutes the most dominating gas species in the thermal runaway of anode/electrolyte at 0% and 100% SOC. This implies that, clarifying the origin of H_2_ evolution is of great significance to understand the rooted triggering factor for thermal runaway. Temperature‐resolved XRD equipped with an in situ heating module is also utilized to analyze the bulk phase change of graphite anode at different SOC. For lithiated graphite anode (100% SOC), the exothermic phase transformation of LiC_6_ (001) to LiC_12_ (002), and LiC_12_ (002) to C (002) occurs at ca. 100 and 200 °C, respectively (Figure 3i). But, even if there are no such exothermic phase transformations for delithiated graphite anode (0% SOC) (Figure 3i), the delithiated graphite anode/electrolyte (0% SOC) still exhibits thermal runaway and the self‐heating happens earlier than lithiated NCM523 cathode/electrolyte (0% SOC). In subsequent, it is not difficult to infer that the thermal runaway of pouch cell is induced by the exothermic reactions related to the reactions of the formed SEI layer on graphite anode. This is also evidenced by the fact that, cycled graphite anode (delithiated state, 0% SOC) reacting with electrolyte shows exothermic peak at *ca*. 80–170 °C, which is not appeared for the uncycled pristine graphite anode and electrolyte, during the synchronous thermal analyzer (STA) test (Figure S6, Supporting Information). Conventionally, SEI layer on graphite anode are typically determined to be consisted of inorganic species (such as LiF, Li_2_CO_3_, Li_2_O, Li_2_C_2_, Li_2_C_2_O_4_, LiOH, etc.) and organic species (such as ROCO_2_Li, (CH_2_OCO_2_Li)_2_, ROLi, CH_3_Li, etc.).^[^ ^22^ ^]^ Although the broken of SEI layer is always identified as the initial stage during the thermal runaway process, but the exothermic reactions related to the thermal decomposition of listed inorganic and organic species are not presented in detail to date. Moreover, the underlying cause for large amounts of H_2_ evolution at graphite anode side is still remained unclear. Therefore, some unknown species must be still unidentified in the SEI layer of graphite anode. In some reports, it is suggested that the graphite anode surface is enriched of hydrogen.^[^ ^13^, ^23^ ^]^ Very recently, our group found the existence of LiH on lithium metal anode surface, and pointed out its influence on anode failure in practical lithium metal batteries.^[^ ^24^ ^]^ Herein, by employing a delicately‐designed deuterium‐oxide (D_2_O) titration device connecting with an on‐line gas analysis mass spectrometry (MS) system (**Figure** **4a**), we unprecedentedly identify the existence of LiH on the surface of the graphite anode. Fully‐lithiated graphite (100% SOC) and fully‐delithiated graphite (0% SOC) are titrated by deuterium‐oxide (D_2_O) with the following guideline reactions: Li*_x_* C_6_ + xD_2_O → xLiOD + C_6_ + x/2 D_2_↑, (x ≤ 1); LiH + D_2_O →LiOD + HD↑. Surprisingly, HD (*m*/*z* = 3) signal is observed for both fully‐lithiated graphite (100% SOC) and fully‐delithiated graphite (0% SOC). In addition, the mole of HD and D_2_ (*m*/*z* = 4) at fully‐lithiated graphite (100% SOC) is 0.14 and 4.26 µmol mg^−1^, respectively (Figure 4b), which decrease to 0.014 and 0.029 µmol mg^−1^, respectively, when the graphite anode is fully‐delithiated (0% SOC) (Figure 4c). It is, the first time, discovered that LiH does exist in the SEI layer of graphite anode, and it exhibits highly electrochemical reversibility at graphite anode surface during cycling. In addition, in another delicately‐designed experiment, fully‐lithiated graphite (100% SOC) is heated at 90 °C, in the titration vessel of abovementioned on‐line gas analysis MS system, H_2_ signal (*m/z* = 2) is detected after heating, confirming that the broken of SEI layer is accompanied by evolution of H_2_ (Figure 4d). Obviously, the released amount of H_2_ (Figure 3e,f) is highly correlated with the determined amount of LiH. Highly lithiated graphite (high SOC) containing more LiH releases more H_2_ when heating. Moreover, LiH was evidenced to have poor thermal compatibility with the dual‐salt electrolyte (Figure 4e), with the exothermic reaction starts at *ca*. 70 °C, which agreed well with the STA results of cycled anode/electrolyte shown in Figure S6 (Supporting Information), implying the critical contribution of LiH/electrolyte exothermic reaction on the early triggering the thermal runaway of the pouch cell. Besides, the presence of LiH is also confirmed in graphite anode disassembled from NCM523/G and NCM811/G pouch cells (using conventional LiPF_6_ based carbonate electrolyte), regardless of SOC, indicating this phenomenon is universal applicable for varied NCM/G cells (Figure S7, Supporting Information). These amazing results indicate that the presence of LiH in graphite anode as well as its active chemical characters during elevated temperature lead to the early heat releasing of the battery, while the phase transformation of lithiated graphite anode and the O_2_‐releasing by delithiated NCM cathode are just accelerating factors for thermal runaway.

**Figure 5:**
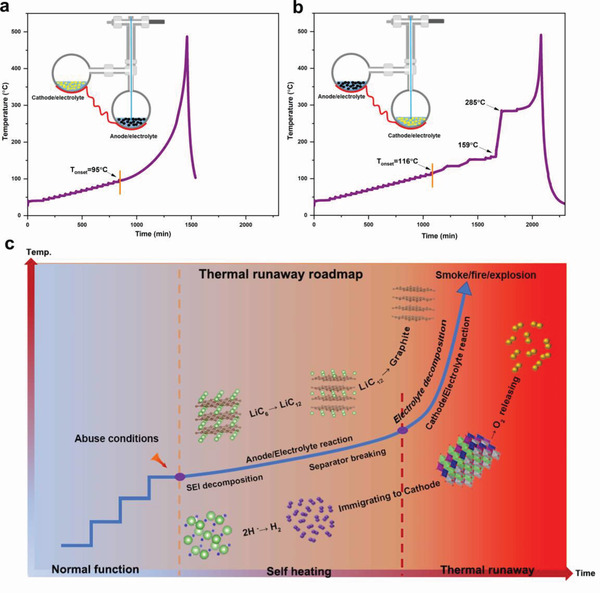
(a) Temperature profile when 100% SOC anode/electrolyte (connected with a bomb chamber containing 100% SOC cathode/electrolyte) are tested by the heat‐wait‐search (HWS) mode of ARC. (b) Temperature profile when 100% SOC cathode/electrolyte (connected with a bomb chamber containing 100% SOC anode/electrolyte) are tested by the heat‐wait‐search (HWS) mode of ARC. Insets are the specific settings. (c) Thermal runaway route map for fully charged NCM/graphite cell.

The aforementioned released gases and temperature profiles during thermal runaway of anode/electrolyte and cathode/electrolyte are separately determined and analyzed, not considering of anode/cathode crosstalk. However, in practical cases, despite anode and cathode is physically separated by the separator, the porosity of separator always allow the crosstalk of byproducts.^[^ ^7^, ^25^ ^]^ Then the question comes: during the triggering process of thermal runway, is there any crosstalk effect when the released gas species migrate through porous separators? To answer this question, a self‐made two bomb chamber testing system is delicately fabricated, where the anode and cathode is placed separately, but the released gas species can flow freely by the connected pipeline, and two bomb chambers are heated by one same heating wire in the quasiadiabatic cavity of ARC (insets in **Figure** **5a**,b; and Figure S8, Supporting Information). Connected with the bomb with fully delithiated NCM523 cathode/electrolyte, the fully‐lithiated graphite/electrolyte reaction delivers a same *T*_onset_ of 95 °C and a lower *T*_tr_ (from 306 to 247 °C) (Figure 5a), compared to the one bomb test of the fully‐lithiated graphite/electrolyte mentioned above (Figure 3e). This clearly tells us that the triggering of anode/electrolyte thermal runaway at low temperatures is not affected by gas species generated by cathode/electrolyte reactions. But at high temperatures, the released gas species (especially O_2_) will accelerate the thermal runaway of anode/electrolyte. Another two‐bomb testing reveals that the gas species (especially H_2_) produced by anode/electrolyte decrease the thermal stability of cathode/electrolyte (Figure 5b). In specific, *T*_onset_ drops from 136 to 116 °C, and a sharp temperature rise from 159 to 285 °C is appeared in the HWS curve. Furthermore, gas species collected from the two‐bomb system (100% SOC electrodes) are mainly consisted of CO_2_ (28.5%), CH_4_ (32.7%), H_2_ (15.2%), and O_2_ (12%), suggesting that H_2_ from anode and O_2_ from cathode side is consumed, and subsequently, evidencing the occurrence of the crosstalk of the released gas species during thermal runaway (Figure S9, Supporting Information). Moreover, H_2_ is preliminarily calculated to have higher affinity to NCM (Figure S10, Supporting Information), suggesting that the released H_2_ has high tendency to react with the NCM in elevated temperatures. In summary, it is concluded here that the LiH induced H_2_ releasing at anode side and H_2_ migration to cathode side is the rooted thermal runaway trigger of NCM523/G pouch cell, while the phase transformation of lithiated graphite anode and the O_2_‐releasing by delithiated NCM523 cathode are just accelerating factors for thermal runaway. Based on all the analysis and experiments, a modified and upgraded thermal runaway route map for 100% SOC NCM523/graphite pouch cell is depicted here (Figure 5c): 1) under abuse (mechanical, electrical, or thermal) conditions, when the battery temperature increases, mild exothermic reactions related to LiH/electrolyte reactions, phase transformation of LiC_6_ to LiC_12_, and SEI layer destruction happens, which is accompanied by H_2_ releasing and corresponding heat releasing; 2) Parts of released H_2_ will diffuse to cathode side, interacting with the fully delithiated NCM to release heat; The reactions in step 1 and step 2 will raise the temperature to ca. 200 °C, at which the polyolefin separators have been melted and partial short circuit between the cathode and anode poles will continue to push up the temperature; 3) when the temperature is pushed up to the range of 200–250 °C, three severe exothermic reactions happens (electrolyte decomposition; phase transformation of LiC_12_ to graphite; and O_2_‐releasing by delithiated NCM523 cathode) and the released large amounts of gases (O_2_, H_2_, CH_4_, CO, C_2_H_4_, etc.) lead to the final severe thermal runaway (smoke, fire, and even explosion).

### 2.3 Heat Generation During Charge–Discharge Operation

Thermal anomalies inside the cell or pack in the absence of proper thermal management can instigate accelerated cell capacity degradation and even hazardous thermal runaway. Therefore, the heat generation accompanied with battery charge–discharge operation is receiving more and more attention. Generally, heat generated from battery cycling can be divided into reversible heat and irreversible heat. Irreversible heat refers to the ohmic heat from the polarization or overpotential of the cell, while reversible heat is determined by the measurement of cell entropic coefficient depending on the intrinsic nature of the electrode materials (relating with the atom arrangement in the crystal lattice).^[^ ^26^ ^]^ Deciphering the heat generation law of a single cell is essential to design and optimize the battery thermal management system, which ensures batteries in a pack or module running in an ideal temperature range. Here, two boundary scenarios of adiabatic and isothermal environment, representing the worst and best case for the heat management, respectively, are considered for heat determination of the pouch cell with dual‐salt electrolyte.

**Figure 6:**
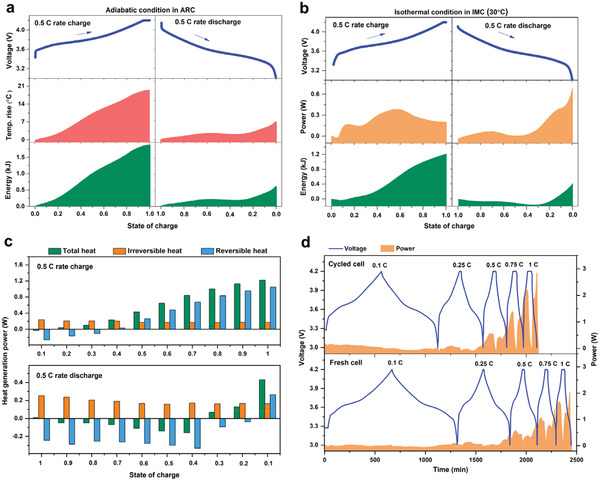
(a) The voltage curve, temperature rise and released energy when charging and discharging 5 Ah NCM523/G pouch cell at 0.5 C rate under the adiabatic mode of ARC equipment (initial temperature 30°C). (b) The voltage curve, heat release power and released energy when charging and discharging 5 Ah NCM523/G pouch cell at 0.5 C rate at 30°C under isothermal condition of IMC equipment. (c) The determined total heat generation power, reversible heat generation power and irreversible heat generation power when charging and discharging 5 Ah NCM523/G pouch cell at 0.5 C rate. (d) The heat generation power of fresh cell and cycled (400 cycles) cell at varied C‐rates.

ARC is a pivotal integrated technology to study the thermal safety of LIBs at multilevel.^[^ ^12^ ^]^ In general, ARC will simulate an accurate adiabatic condition by keeping the cavity temperature consistent with the sample temperature, preventing the self‐generated heat loss of sample. Therefore, ARC is the worse‐case scenario. Here, the 5 Ah NCM523/G pouch cell with the dual‐salt electrolyte is placed in the adiabatic cavity of ARC (BTC500, HEL, Figure S11, Supporting Information) and connected with a charge/discharge apparatus. Obviously, the surface temperature of pouch cell increases during both charge and discharge process (**Figure** **6a**; and Figure S12a–c, Supporting Information). For example, at 0.5 C rate, the overall heat generation (19.5 °C, 1.9 kJ) during the charge process is much higher than that (6.3 °C, 0.6 kJ) of the discharge process. At varied C‐rates, the self‐heating rate curves during charge and discharge process are symmetrical (Figure S12d–f, Supporting Information), evidencing that the heat generation mainly consisted of the irreversible joule heat and the reversible electrochemical reaction heat.^[^ ^27^ ^]^ Obviously, the reversible electrochemical reaction heat dominates the total heat at low rates and irreversible joule heat dominates the total heat at high rates. These results clearly tell us that, at the worst case of adiabatic condition, the 5 Ah NCM523/G pouch cell at higher rates will easily get thermal runaway in few cycles, revealing the importance of battery thermal management. As for the battery thermal management, it is necessary to determine the heat generation of a single cell at a constant temperature. Here, as a best‐case scenario, IMC (isoBTC, HEL) is used to test the heat generation of the 5 Ah NCM523/G pouch cell at the isothermal condition (Figure S13, Supporting Information). At 0.5 C rate at 30 °C, the overall heat generation (1.2 kJ) during the charge process is much higher than that (0.4 kJ) of the discharge process (Figure 6b). Clearly, the released energy during 1 charge–discharge cycle at isothermal condition (1.6 kJ) is lower than that at the adiabatic condition (2.5 kJ). Interestingly, at 0.5 C rate, more heat is generated at both 10 and 50 °C when compared to 30 °C, suggesting the importance of selecting work temperature for battery (Figure S14, Supporting Information). The reversible and irreversible heat are determined based on the ohmic resistance and total heat generation.^[^ ^28^ ^]^ The internal resistance of the pouch cell is calculated by the hybrid pulse power characterization method (Figure S15, Supporting Information). On the whole, the reversible heat dominates the total heat generation at 0.5 C rate, and the endothermic and exothermic reactions are distinguishable and transformable during the charge or discharge process (Figure 6c). Compared to fresh pouch cell, long‐term (400 cycles) cycled pouch cell demonstrates much higher heat release power at higher rates, especially at 1 C rate (Figure 6d). In summary, the design of an efficient and smart battery thermal management system must comprehensively consider the effects of work temperature, state of charge (SOC), charge–discharge current rate, and charge–discharge protocol on heat generation.

## 3 Conclusion

In this paper, a LiTFSI‐LiDFOB based dual‐salt electrolyte has been successfully and unprecedentedly demonstrated to possess high compatibility with a high‐energy (208.8 Wh kg^−1^) 5 Ah NCM523/G pouch cell. This pouch cell delivers excellent electrochemical performances over a wide temperature range (−40–60 °C). In subsequent, the thermal safety characteristics of this high‐energy pouch cell is investigated. More importantly, by varied advanced characterization techniques, it is innovatively proposed that the LiH‐induced exothermic reactions at anode side and H_2_ migration to cathode side is the rooted thermal runaway trigger of NCM523/G pouch cell, while the phase transformation of lithiated graphite anode and the O_2_‐releasing by delithiated NCM523 cathode are just accelerating factors for thermal runaway. Heat determination under adiabatic condition states the necessity of designing battery thermal management system, while heat determination under isothermal condition reveals that an efficient and smart battery thermal management system must comprehensively consider the effects of work temperature, SOC, charge–discharge current rate, and charge–discharge protocol on heat generation. These findings will shed promising lights on thermal runaway prevention as well as development of high energy safe LIBs.

## 4 Experimental Section

### Pouch Cell and Electrolyte

Dry (no electrolyte injection) 5 Ah LiNi_0.5_Co_0.2_Mn_0.3_O_2_/graphite (NCM523/G) pouch cells with gas pocket were manufactured by Shandong Kingpin Energy Co., Ltd., China. All battery grade lithium salts (LiPF_6_, LiDFOB, and LiTFSI) and solvents (EC, PC, and EMC) were purchased without purification (Suzhou Qianmin Chemistry Co., Ltd., China). The dual‐salt electrolyte was formulated by dissolving 0.6 M LiTFSI and 0.4 M LiDFOB in carbonate solvents of EC/PC/EMC (1:1:3, by volume), and 0.05 M LiPF_6_ was used as functional additive. For comparison, 1.0 M LiPF_6_ EC/PC/EMC (1:1:3, by volume) was also prepared. All electrolytes were prepared in an argon‐filled glovebox (Mikrouna, China) with H_2_O and O_2_ less than 1 ppm.

### Electrochemical Measurements over Wide Temperature Range

The 5 Ah NCM/G pouch cells were charged and discharged using a LAND battery testing system (Wuhan LAND electronics Co., Ltd. China). All pouch cells were charged and discharged at 0.1 C at room temperature for the one formation cycle, then vacuum degassed to remove gas pocket. For room temperature cycling performance at 0.5 C, all pouch cells were charged to 4.2 V, followed by a constant potential step at 4.2 V for 10 min, then discharged to 3.0 V. For 60 °C cycling performance at 0.2 C, all pouch cells were charged to 4.2 V, followed by a constant potential step at 4.2 V for 10 min, then discharged to 3.0 V. For low temperature discharge performance, all pouch cells were charged, with 0.5C, to 4.2 V at 0.2 C, followed by a constant potential step at 4.2 V for 10 min at room temperature (25 °C), then discharged to 3.0 V at 0.1 C at varied subzero temperatures of −10, −20, −30, and −40 °C.

### Study of Thermal Runaway Feature and Mechanism

Thermal runaway study of 5 Ah NCM/G pouch cell was conducted using an ARC BTC500 (HEL, England), which was equipped with a digital camera (see Figure S1, Supporting Information). Here, typical HWS mode was adopted for testing: one heating step was 5 °C; detection limit was 0.03 °C min^−1^; the temperature was raised from 40 to 250 °C. The thermal runaway criteria is 1 °C min^−1^.

Thermal compatibility study of battery materials was investigated by a small standard ARC (BTC130, HEL, England, see Figure 4a; and Figure S4, Supporting Information). The anode and cathode are carefully dissembled from fully charged (100% SOC) and fully discharged (0% SOC) 5 Ah NCM523/G pouch cells, in an argon‐filled glovebox. The electrode materials were carefully removed from the current collector with a surgical blade. 0.5 mL electrolyte and 1 g electrode material were transferred into the small bomb chamber made of Hastelloy alloy. Here, typical HWS mode was adopted for testing: one heating step was 5 °C; detection limit was 0.03 °C min^−1^; the temperature was raised from 40 to 250 °C. The thermal runaway criteria is 1 °C min^−1^. When the small bomb chamber was cooled down after thermal runaway, the released gases was collected to be analyzed by a mass spectrum (MS, HPR‐20, Hiden Analytical Ltd.).

The thermal stability of battery materials was also tested in a Simultaneous Thermal Analyzer (STA, Netzsch): heating rate was 5 °C min^−1^; the temperature was raised from 25 to 500°C. Temperature‐resolved X‐ray diffraction XRD (with Cu K*α* radiation, *λ* = 1.5406 Å) was tested on a Ultima IV of Rigaku: the sample was sealed in an Ar‐filled container; the angle ranged from 10°to 80°with a scan speed of 20° min^−1^; the temperature was raised from 30°C to 400°C with a heating rate of 1°C min^−1^. Thermal compatibility of LiH with electrolyte was testing by DSC under N_2_ atmosphere, with the temperature of 30°C to 400°C and the ramp rate of 5°C min^−1^. All the samples were prepared in an argon‐filled glovebox (Mikrouna, China) with H_2_O and O_2_ less than 1 ppm.

The on‐line gas analysis system was used to real‐time monitor the gas produced from the titration system, which is mainly composed of carrier gas cylinder, self‐made titration unit, and mass spectrometer (HPR‐20, Hiden Analytical Ltd.). During the D_2_O titration experiments, 10 mg graphite anode with 0% SOC or 100% SOC was placed in the titration unit and 2 mL D_2_O was injected, and the liberated gases are analyzed by the mass spectrometer. For quantitative analysis, the intensity signal of D_2_, HD gas was divided by the intensity of the carrier gas (Ar), the resulting ratio was multiplied by the flow rate (1.5 mL min^−1^) and divided by the sample mass to get the flow rate per unit mass (adopting V_m_ = 24.5 L mol^−1^, 25°C, 101 KPa).

After connecting the sample‐containing titration unit to the *on‐line* gas analysis system, the Ar carrier gas was switched to go through the titration unit, then 1 ml D_2_O was injected and the gas species (mainly D_2_ and HD) was recorded by the (MS HPR‐20, Hiden Analytical Ltd.). In another test, the unit containing the 100% SOC graphite anode was heated to 90°C, and the released gas species were detected (See Figure 4).

To study the crosstalk effects of the released gas species, a self‐made bomb chamber testing system is delicately fabricated (Figure S8). Two separated bomb chambers were connected by one pipeline, where the gases could exchange while the solid/liquid reactants keep uncontacted. During the test, the anode/electrolyte and cathode/electrolyte are placed separately in these two bombs, and the two bombs were heated by the same heating wire during the heating process. As the temperature increase, the released gas species from one bomb chamber can flow freely to the other one by the connected pipeline, thus the crosstalk of the gases could be detected.

### Heat Generation at Adiabatic Condition

For heat generation determination at adiabatic condition, the 5 Ah NCM/G pouch cells were put inside the ARC cavity (BTC500, HEL, England) and connected with a charge‐discharge device (Wuhan LAND electronics Co., Ltd. China) (See Figure S11). The adiabatic test mode of ARC is used, and three different current rate of 0.25 C, 0.5 C, and 1 C were used to investigate the temperature increase and self‐heating rate of the pouch cell during the charge and discharge process. After each charge or discharge process, the cavity temperature of ARC was cooled back to the initial temperature of 30°C.

### Heat Generation at Isothermal Condition

Isothermal heat generation during charge and discharge process was determined by an isothermal battery calorimeter (IMC, iso‐BTC, HEL, England). The tested pouch cell was enclosed by graphite film with high heat conductivity. Heating sheets were fixed on graphite film (fixed on the side close to pouch cell), and power sensor was fixed on pouch cell (See Figure S13). The pouch cell was tested at 0.5 C at varied temperatures of 10°C, 30°C, and 50°C, respectively, and the corresponding total specific heat generation rate (*q*) was determined.

The total specific heat generation rate (*q*) included the reversible heat generation rate (*q_r_*) and irreversible heat generation (*q_i_*). *q* was determined by the IMC testing, and *q_i_* is calculated by I^2^R, where I is the working current, and R is the total internal resistance. The reversible heat generation rate (*q_r_*) was obtained by *q_r_* = *q* – *q*_i_. The total internal resistance R of the pouch cell was determined by the hybrid pulse power characterization using the battery test system. The pouch cell was adjusted to a specific SOC (10%, 20%, 30%, 40%, 50%, 60%, 70%, 80%, 90%, and 100%), and then the following protocol was used: 1 C rate pulse for 10 s discharge, followed with a 40 s rest, then a 0.75 C pulse was used to charge for 10 s. Each SOC adjustment was followed by a one‐hour rest period. Finally, the total internal resistance R during charge and discharge was calculated as mentioned previously.^[^ ^28^ ^]^

## Conflict of Interest

The authors declare no conflict of interest.

## Author Contributions

L.H. and G.X. contributed equally to this work. L.H. Investigation, Writing – original draft. G. X., Methodology, Writing – review & editing. X. D., Software. J. L., Formal Analysis. B. X., Validation. H. L., Resources. P. H., Formal Analysis, Funding acquisition. S.D., Writing – review & editing. G. C., Conceptualization, Funding acquisition, Supervision. L. C. Validation, Writing – review & editing.

## Supporting information

Supporting Information

## Data Availability

The data that support the findings of this study are available from the corresponding author upon reasonable request.
