# Supplementary material for: Uncovering LiH Triggered Thermal Runaway Mechanism of a High‐Energy LiNi0.5Co0.2Mn0.3O2/Graphite Pouch Cell
Source: Adv Sci (Weinh). 2021 May 24;8(14):2100676. doi: 10.1002/advs.202100676 (PMC8292879; doi:10.1002/advs.202100676)
Supplement: Supplementary file 1 — Supporting Information [file ADVS-8-2100676-s001.pdf]

© 2021 Wiley-VCH GmbH

ADVANCED SCIENCE
Open Access

Supporting Information

for *Adv. Sci.*, DOI: 10.1002/advs.202100676

Uncovering LiH triggered thermal runaway mechanism of a high-energy $LiNi_{0.5}Co_{0.2}Mn_{0.3}O_2$/graphite pouch cell

Lang Huang, Gaojie Xu, Xiaofan Du, Jiedong Li, Bin Xie, Haisheng Liu, Pengxian Han, Shanmu Dong, Guanglei Cui, Liquan Chen*

Uncovering LiH triggered thermal runaway mechanism of a high-energy $LiNi_{0.5}Co_{0.2}Mn_{0.3}O_2$/graphite pouch cell

Lang Huang[1], Gaojie Xu[1,2], Xiaofan Du[1], Jiedong Li[1], Bin Xie[1], Haisheng Liu[1], Pengxian Han[1], Shanmu Dong[1], Guanglei Cui[1,*], Liquan Chen[2,3]

[1]Qingdao Industrial Energy Storage Research Institute, Qingdao Institute of Bioenergy and Bioprocess Technology, Chinese Academy of Sciences, No. 189 Songling Road, Qingdao 266101, China.

[2]Center of Materials Science and Optoelectronics Engineering, University of Chinese Academy of Sciences, No. 19A Yuquan Road, Beijing 100049, China.

[3]Key Laboratory for Renewable Energy, Beijing Key Laboratory for New Energy Materials and Devices, Beijing National Laboratory for Condensed Matter Physics, Institute of Physics, Chinese Academy of Sciences, Beijing 100190, China

[*]Correspondence: cuigl@qibebt.ac.cn

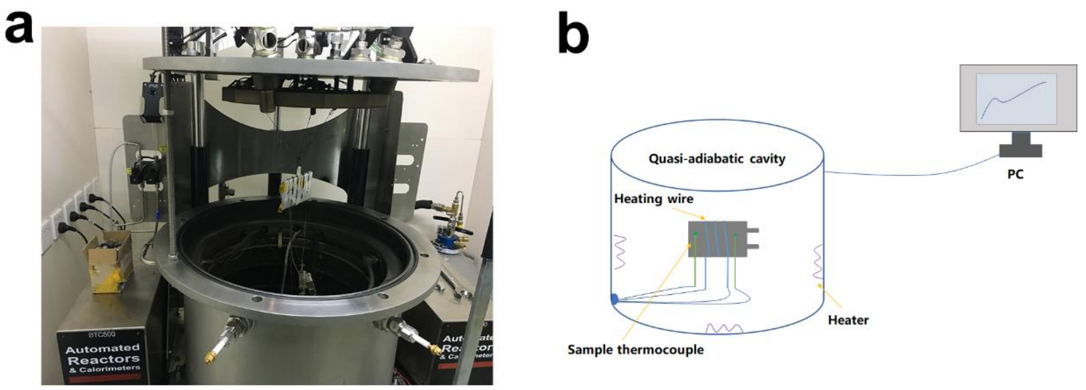

Figure S1 (a) The photographs of ARC (BTC500, HEL) for thermal runaway study. (b) Schematic illustration of the working principle.

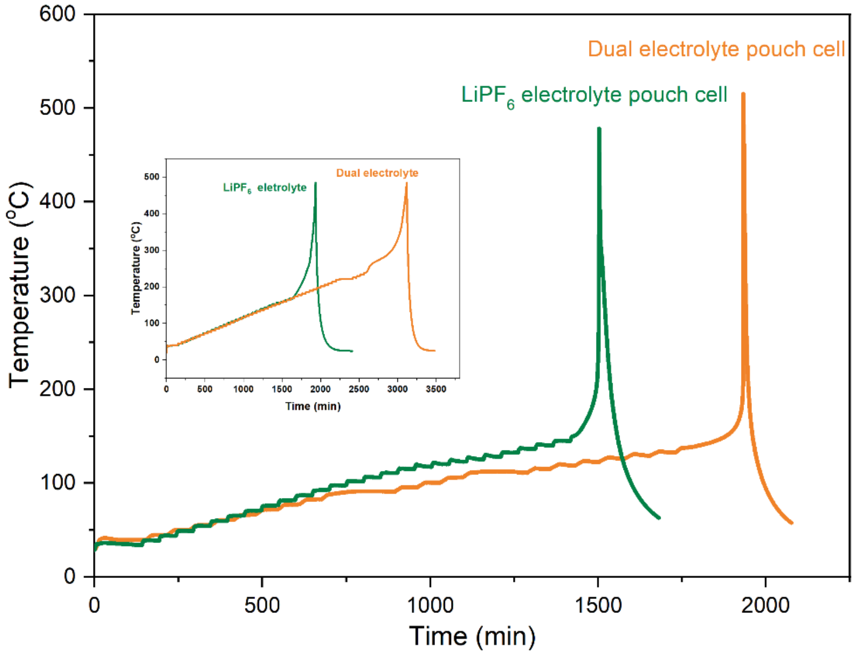

Figure S2 Thermal stabilities of $LiPF_6$ and dual-salt electrolyte and their thermal runaway curves.

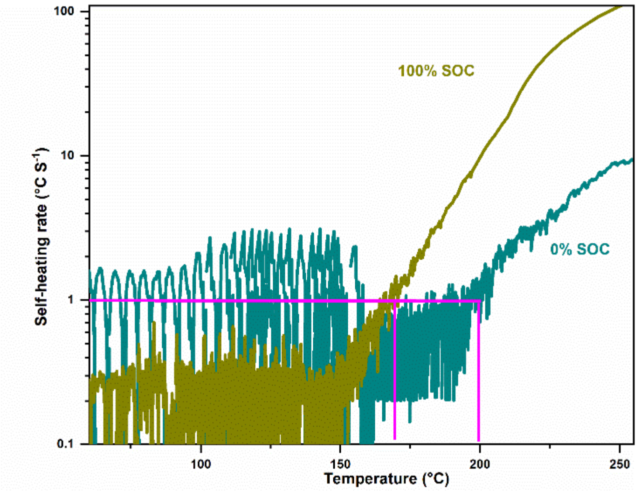

Figure S3 The self-heating rate curves when 5 Ah NCM523/G pouch cells (at 0% SOC and at 100% SOC, after formation process) are tested by the heat-wait-search (HWS) mode of ARC.

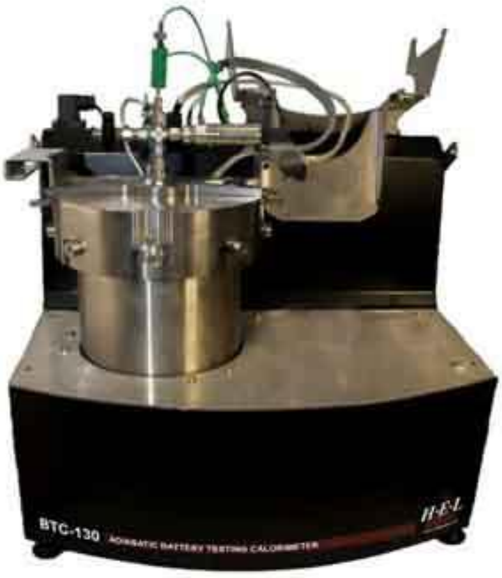

Figure S4 The photographs of ARC (BTC130, HEL) for thermal runaway study of battery materials.

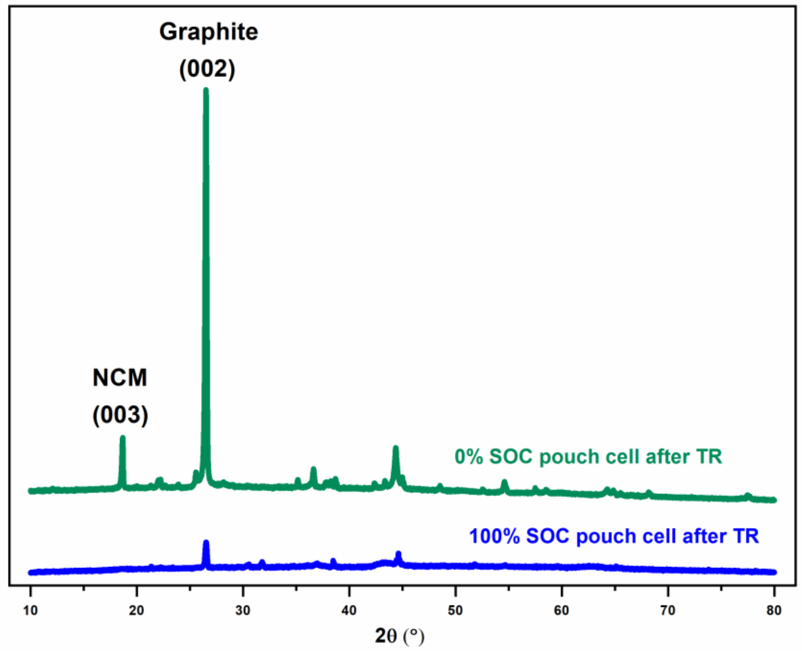

Figure S5 XRD patterns of powders collected after thermal runaway of NCM523/G pouch cell (0 and 100% SOC).

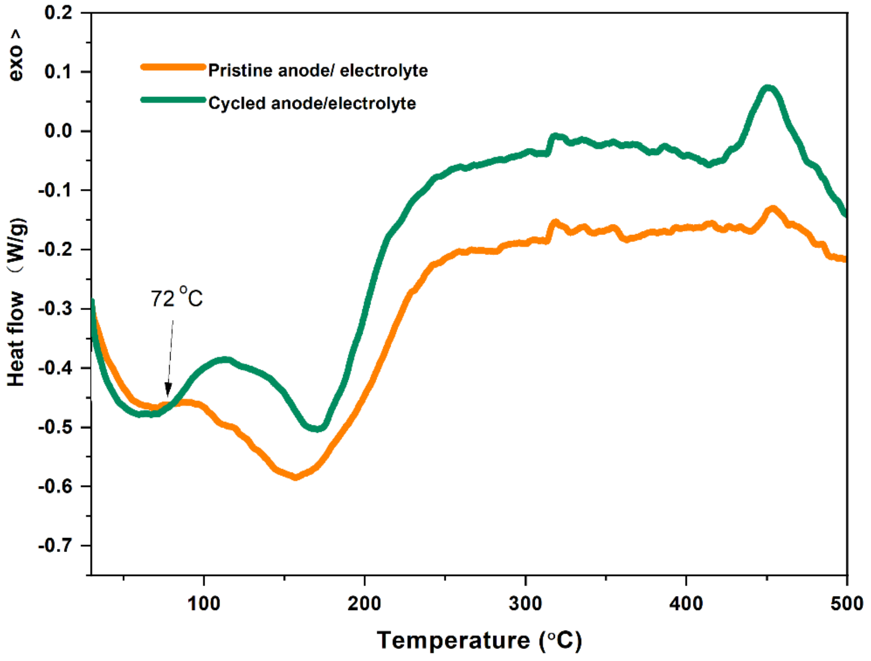

Figure S6 Heat flow of pristine anode/electrolyte, and cycled anode (0% SOC) /electrolyte when tested in a STA system.

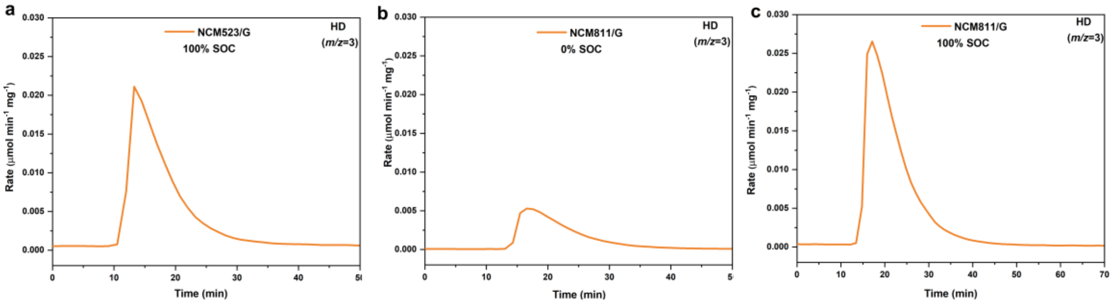

Figure S7 The HD evolution rate curve after D_2O titration on samples of (a) fully-lithiated graphite dissembled from fully charged (100% SOC) NCM523/G pouch cell using commercial $LiPF_6$ carbonate electrolyte, (b) fully-delithiated graphite from fully discharged (0% SOC) NCM811/G pouch cell using commercial $LiPF_6$ carbonate electrolyte, and (c) fully-lithiated graphite dissembled from fully charged (100% SOC) NCM811/G pouch cell using commercial $LiPF_6$ carbonate electrolyte.

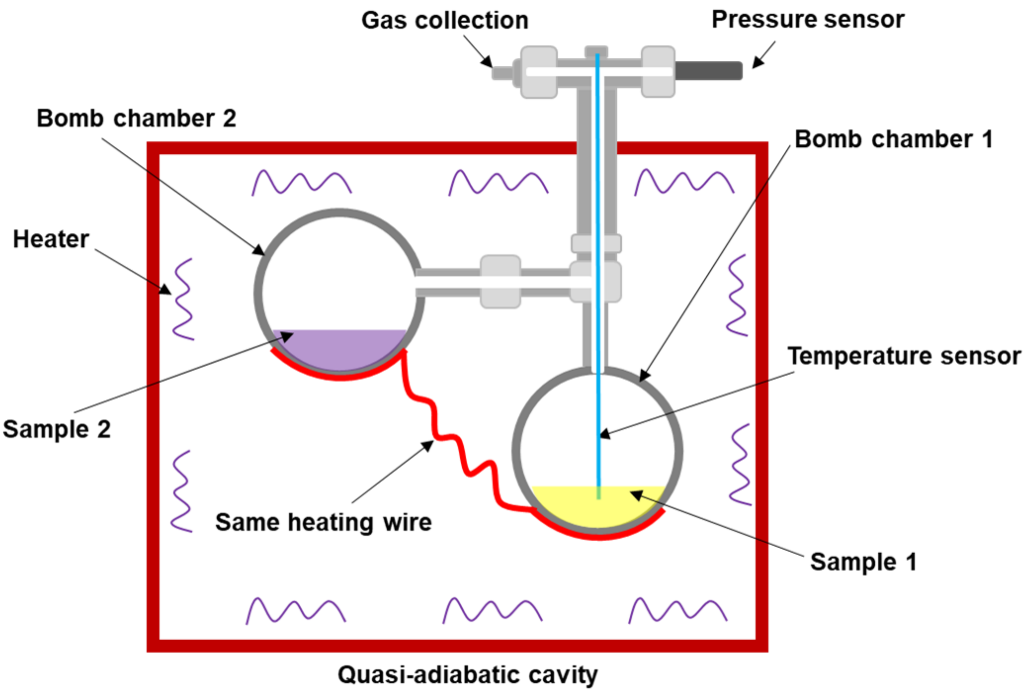

Figure S8 Schematic illustration of a self-made system containing two bomb chambers, for crosstalk effect study in ARC (BTC130, HEL).

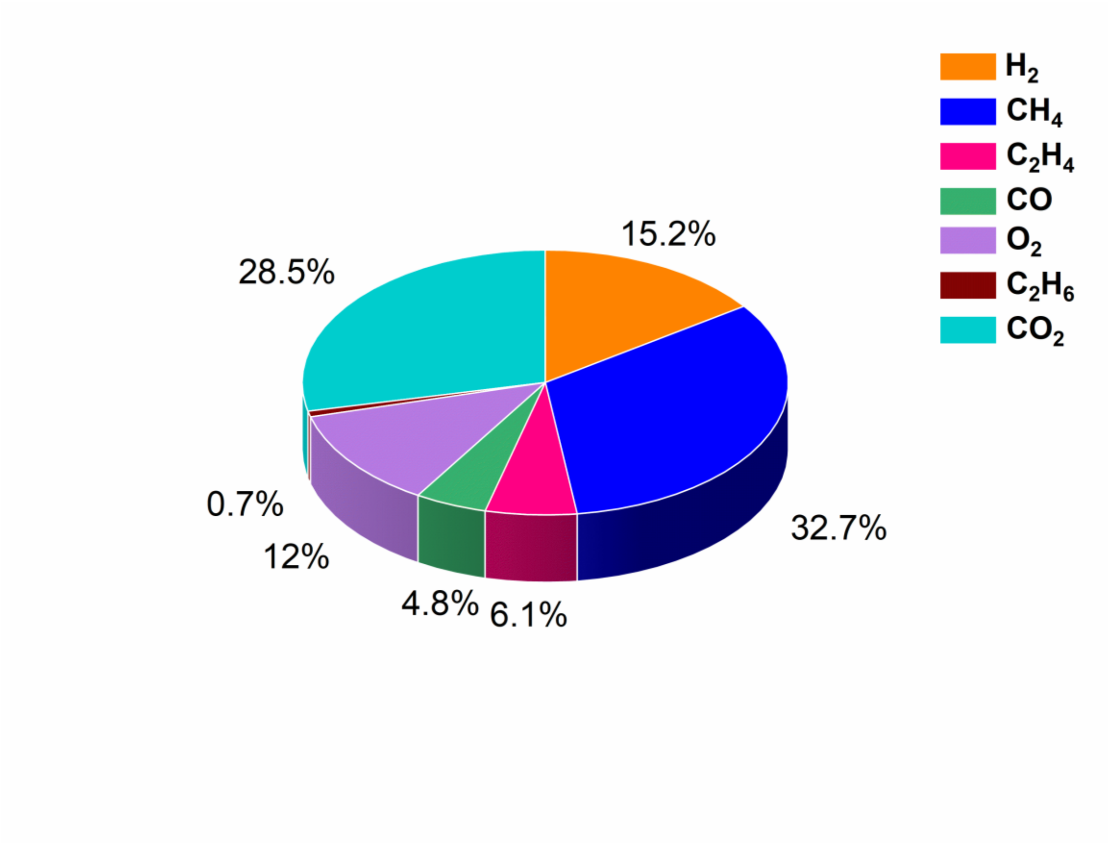

Figure S9 The determined components percentage in collected gas after ARC testing, in a self-made two bomb chamber system containing cathode/electrolyte and anode/electrolyte separately (as illustrated in Figure S8)

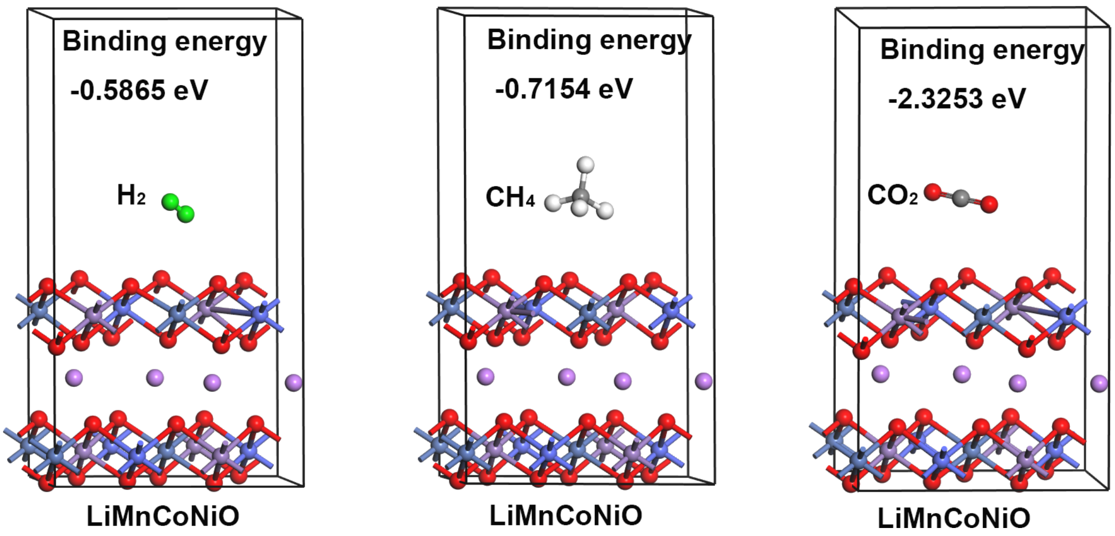

Figure S10 The calculated binding energy of H_2, CH_4, and CO_2 with NCM cathode

Computational methods

Periodic planewave DFT+U calculations for the cathode interface systems were performed using the Vienna ab initio Simulation (VASP) [1-3] within MedeA® computational environment [4] with the spin-polarized Perdew-Burke-Ernzerhof (PBE) exchange-correlation functional and the projector-augmented wave (PAW) scheme to treat core electrons [5-7]. VASP standard pseudopotentials (PP) were used for carbon, oxygen, and hydrogen atoms. PAW PPs employed for the transition metal atoms Co, Ni and Mn (denoted as Co_pv, Ni_pv and Mn_pv, respectively) treat p semi-core states as valence states. The Li_sv PP used for Li treats the 1s shell as valence states. For the +U augmented treatment of Co, Ni and Mn 3d orbitals, we chose a U_{eff} value of 4.91eV for Co, 5.96 eV for Ni and 5.0 eV for Mn [8, 9]. To describe the weak van der Waals'force (VDW), the DFT-D3-BJ-damping correction was adopted for geometric optimization [10]. We adopted the NCM disordered configuration (Monoclinic phase) with distributed Co, Ni and Mn ions. The lattice parameters, cell volumes and the atomic positions are fully optimized and relaxed until the energy is less than 1×10^{-5} eV per unit cell and the force on each atom is less than 0.02 eV $Å^{-1}$. We used the conjugate gradient minimization method for geometry optimization. For our simulated structures, the kinetic energy cutoffs were set at 500 eV for

the plane wave basis set and the calculation were spin-polarized. A Monkhorst-Pack grid with 2×2×2 meshes was employed in the irreducible Brillouin zone [11]. The (001) surface for the fully delithiated NCM structure was generated by removing the two Li atoms from the corresponding fully lithiated slab structure and re-optimizing. The NCM (001) slab consists of 7 layers and an xy-plane of dimensions 5.0 Å x 8.6 Å with 15 Å of vacuum in the z-direction. In order to calculate the adsorption energies of gas molecules on NCM (001) slab, we use the following formula:

$$E_{ads} = E_{tot} - E_{sheet} - E_{gas\ molecules}$$

where E_{tot}, E_{sheet} and $E_{gas\ molecules}$ represents the total energy of the system, energy of NCM (001) slab and energy of gas molecules.

References:

[1] G. Kresse, J. Hafner. Phys. Rev. B 1993, 47, 558

[2] G. Kresse, J. Furthmüller. Phys. Rev. B 1996, 54, 11169

[3] G. Kresse, J. Joubert. Phys. Rev. B 1999, 59, 1758

[4] MedeA® version 3.02, 2020, Materials Design, Inc., Santa Fe, NM 87501

[5] J.P. Perdew, K. Burke, M. Ernzerhof, Phys. Rev. Lett., 77 (1996) 3865-3868.

[6] J.P. Perdew, K. Burke, M. Ernzerhof, Phys. Rev. Lett., 1997, 78: 1396-1396.

[7] G. Kresse, D. Joubert, Phys. Rev. B, 59 (1999) 1758-1775.

[8] F. Zhou, M. Cococcioni, C.A. Marianetti, D. Morgan, G. Ceder, Physical Review B, 70 (2004).

[9] G. Zhong, Y. Li, P. Yan, et al. J.Phys. Chem. C, 2010, 114(8), 3693–3700.

[10] J.Klimeš, D. R. Bowler, A. Michaelides, Phys. Rev. B: Condens. Matter Mater. Phys. 2011, 83, 195131.

[11] F. Du, X. Ren, J. Yang, J. Liu and W. Zhang, J. Phys. Chem. C, 2014, 118, 10590–10595

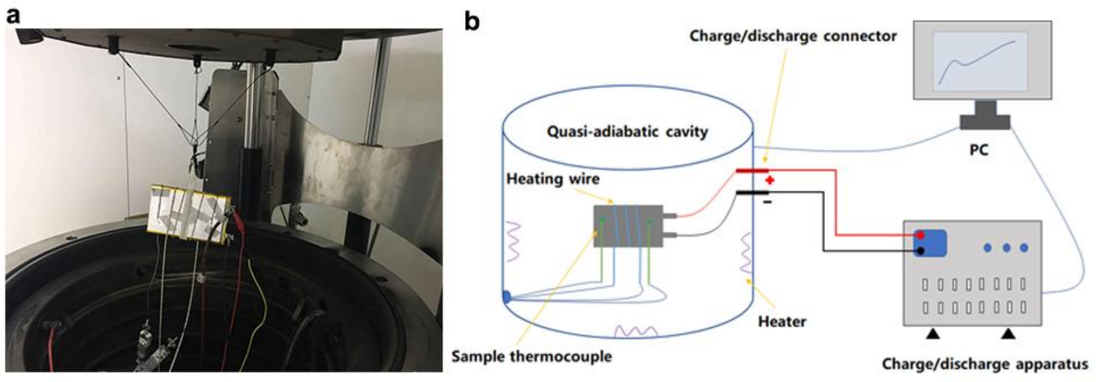

Figure S11 (a) The photographs of ARC (BTC500, HEL) for total heat determination. (b) Schematic illustration of the working principle.

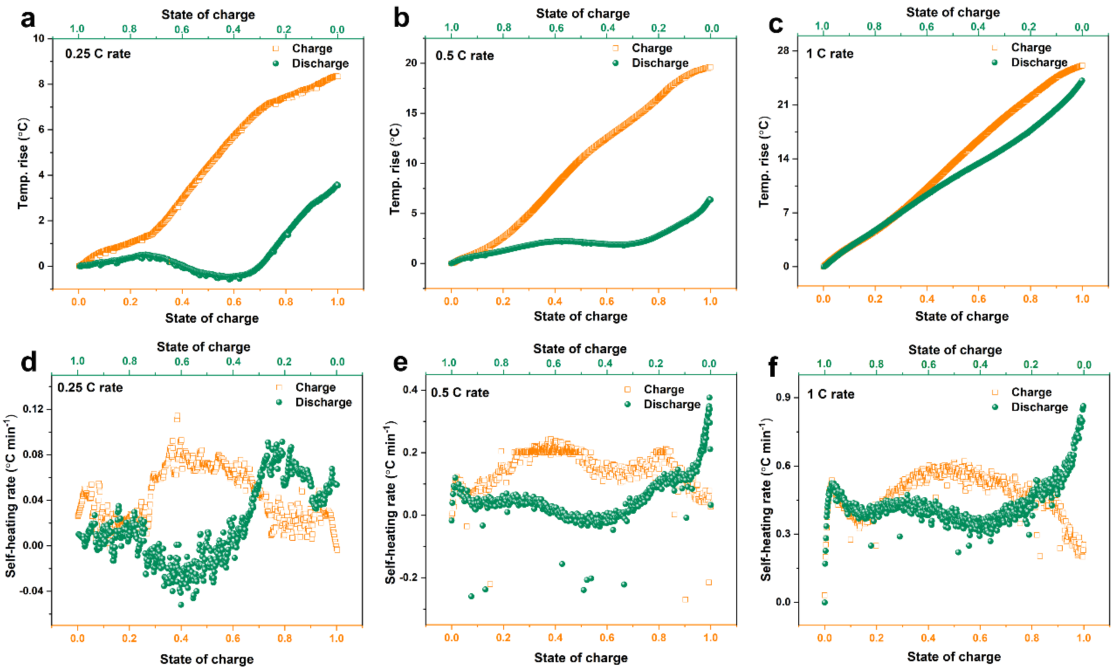

Figure S12 (a-c) The temperature rise and (d-f) self-heating rate when charging and discharging 5 Ah NCM523/G pouch cell at varied current rates under the adiabatic mode of ARC equipment.

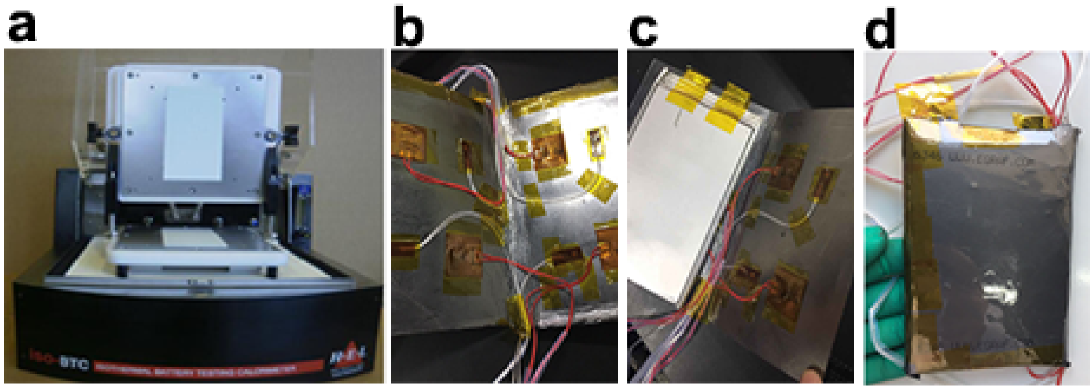

Figure S13. (a) The photographs of isoBTC (HEL). (b-d) Photographs showing the set-up of pouch cell for isothermal testing.

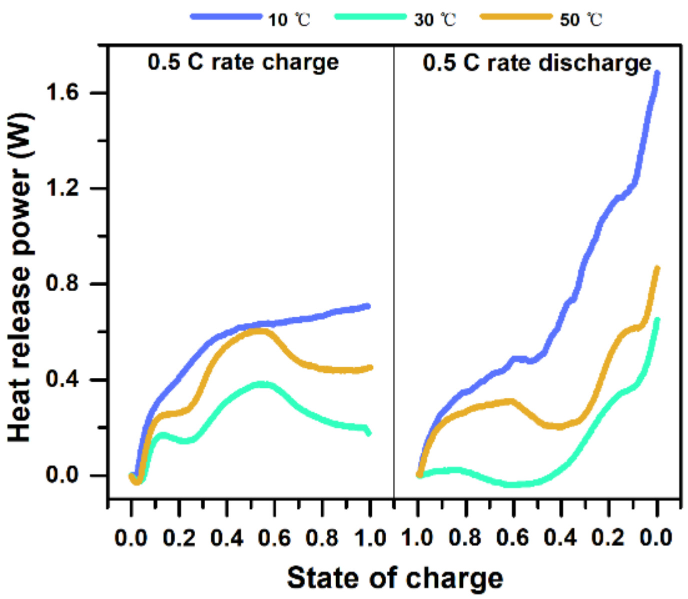

Figure S14. The heat release power when charging and discharging 5 Ah NCM523/G pouch cell at 0.5 C rate at varied temperature of 10℃, 30℃, and 50℃, under isothermal condition of IMC equipment.

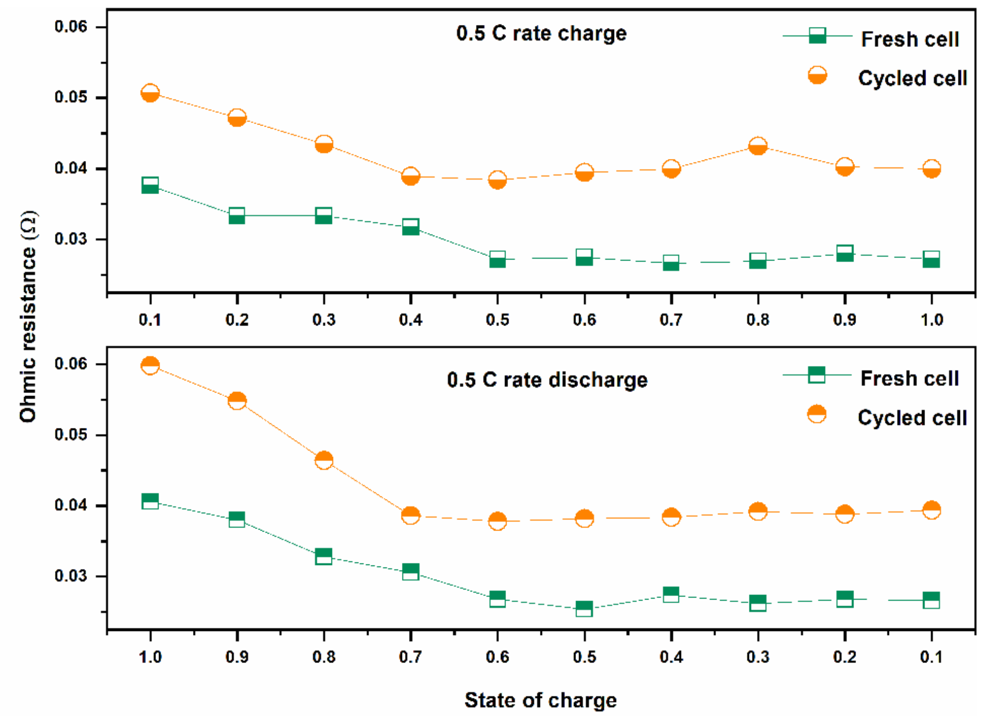

Figure S15. The internal ohmic resistance of 5 Ah NCM523/G pouch cell (fresh cell and cycled cell for 400 cycles) at 0.5 C rate
